# Supplementary material for: Silicon Supply Improves Nodulation and Dinitrogen Fixation and Promotes Growth in Trifolium incarnatum Subjected to a Long-Term Sulfur Deprivation
Source: Plants (Basel). 2023 Jun 8;12(12):2248. doi: 10.3390/plants12122248 (PMC10303559; doi:10.3390/plants12122248)
Supplement: Supplementary file 1 [file plants-12-02248-s001.zip › plants-2441465-supplementary.pdf]

Supplementary Materials:

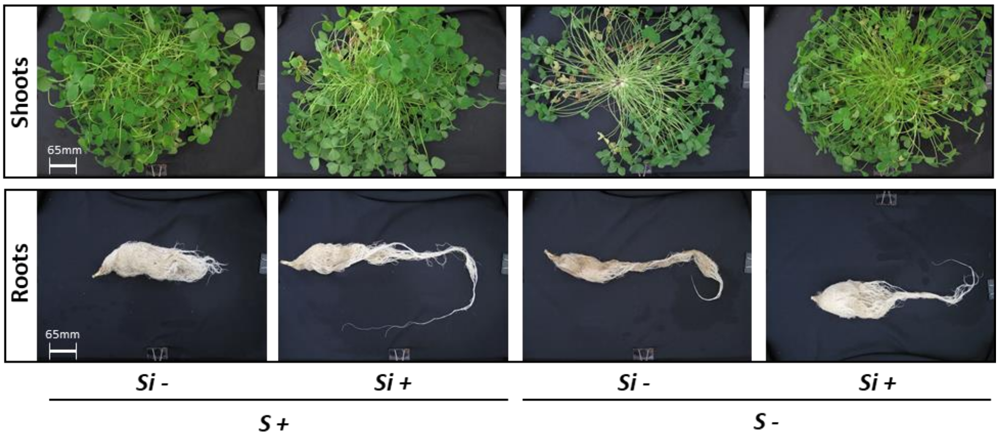

Figure S1. Representative pictures of roots and shoots of *Trifolium incarnatum* plants cultivated in hydroponic conditions at 63 days (D63) with (+S; 500 µM) or without (-S) sulphur and with (+Si; 1.7 mM) or without (-Si) Si supply.

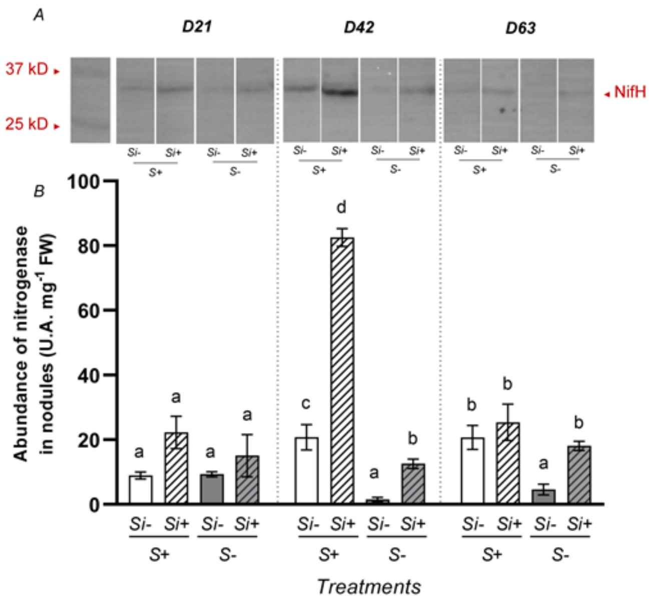

Figure S2: Change in nitrogenase abundance in nodules of *Trifolium incarnatum* plants cultivated in hydroponic conditions during 21 (D21), 42 (D42) and 63 days (D63) with (+S; 500 µM) or without (–S) sulphur and with (+Si; 1.7 mM) or without (-Si) Si supply. Representative western blot performed with specific antibody of nitrogenase (NifH) (*A*). At each harvest date and for each treatment, the quantification of nitrogenase abundance was expressed to the fresh weight of the nodules (*B*). Data are means ± SE (n=3). Different Latin letters indicate that the mean of comparison of nitrogenase relative amount between plants harvested at the same time (D21, D42, D63) are significantly different ($p < 0.05$).
